# Functional and Biochemical Characterization of Spermidine Synthase CauSpe3 from *Candidozyma auris*

**DOI:** 10.3390/pathogens15040432

**Published:** 2026-04-16

**Authors:** Jae-Yeon Choi, Pallavi Singh, Choukri Ben Mamoun

**Affiliations:** 1 Department of Internal Medicine, Section of Infectious Diseases, Yale School of Medicine, New Haven, CT 06520, USA; jae-yeon.choi@yale.edu (J.-Y.C.); pallavi.singh@yale.edu (P.S.); 2 Department of Microbial Pathogenesis, Yale School of Medicine, New Haven, CT 06520, USA; 3 Department of Pathology, Yale School of Medicine, New Haven, CT 06520, USA

**Keywords:** putrescine, spermidine, spermine, polyamines, fluorescence assay, 1,2-diacetyl benzene, enzyme activity, kinetics, yeast, drug discovery, inhibition

## Abstract

Polyamines, putrescine, spermidine and spermine, are essential polycationic metabolites present in all eukaryotic cells, where they regulate fundamental processes including nucleic acid stabilization, translation, and stress responses. Spermidine synthase (SPDS), a member of the aminopropyltransferase (APT) family, catalyzes the transfer of an aminopropyl group from decarboxylated S-adenosylmethionine (dc-SAM) to putrescine to form spermidine. Although genomic analyses predict the presence of SPDS homologs in multiple fungal species, polyamine biosynthesis has not been experimentally characterized in the multidrug-resistant fungal pathogen *Candidozyma auris*. Here, we report the biochemical and functional characterization of the *C. auris* spermidine synthase, CauSpe3. The *CauSPE3* gene complemented a *Saccharomyces cerevisiae spe3Δ* mutant demonstrating conserved function *in vivo*. Recombinant CauSpe3 was expressed in *Escherichia coli*, purified and analyzed using the fluorescence-based DAB-APT assay, which uses 1,2-diacetylbenzene (DAB) for polyamine detection. CauSpe3 catalyzed efficient conversion of putrescine to spermidine in the presence of dc-SAM, with *K_half_* values of 65.5 ± 7.11 µM for putrescine and 66.9 ± 2.09 µM for dc-SAM, and *V_max_* values of 7.1 ± 0.57 and 7.9 ± 0.12 nmol·µg^−1^·min^−1^, respectively. A catalytic-site mutant and heat-inactivated enzyme showed no detectable activity, and product formation was confirmed by means of thin-layer chromatography and mass spectrometry. These findings establish CauSpe3 as a functional spermidine synthase.

## 1. Introduction

The fungal pathogen *Candidozyma auris* (formerly known as *Candida auris*) has emerged over the past decade as a major global health threat, causing outbreaks of invasive fungal infections in healthcare settings worldwide [1,2,3]. Of particular concern is this pathogen’s resistance to multiple classes of clinically important antifungal drugs. Equally alarming, the number of reported cases resistant to echinocandins, the first-line therapy for *C. auris* infection, tripled in 2021 [1,3,4]. Individuals who are critically ill, have invasive medical devices, or experience prolonged or repeated stays in healthcare facilities are at increased risk for acquiring *C. auris* [1]. As a result, in March 2023, the Centers for Disease Control and Prevention (CDC) declared *C. auris* an urgent antimicrobial resistance (AR) threat [2]. Another notable feature of this pathogen is its ability to persist on abiotic surfaces, making it difficult to eliminate once introduced into clinical environments [5,6]. This persistence is mediated by a *C. auris*–specific adhesin, Surface Colonization Factor 1 (Scf1), which promotes adhesion to abiotic and biological surfaces across isolates from all five clades, together with the conserved adhesin Iff4109 [7,8]. Despite these challenges, the biology and metabolism of *C. auris* remain poorly understood, and the identification of new therapeutic targets is urgently needed.

For most fungal pathogens, including *C. auris*, traditional antifungal therapies, including azoles, polyenes, and echinocandins, primarily target the fungal cell membrane (ergosterol synthesis or ergosterol itself) or cell wall (ß-1,3-glucan synthesis), pathways that are increasingly compromised by resistance [6,9]. Alternative therapeutic strategies are aimed at targeting essential metabolic pathways that are conserved among fungal pathogens but sufficiently divergent from their human counterparts to enable the development of selective inhibitors. One such target is the fungal pantothenate kinase (PanK), the first and committed enzyme in coenzyme A (CoA) biosynthesis, which controls intracellular CoA levels and downstream metabolic pathways and thereby regulates multiple essential cellular processes, including fatty acid synthesis, ergosterol biosynthesis, and protein acetylation [10,11,12,13]. Other core metabolic pathways targeted by antifungal drug-discovery programs include pyrimidine [14,15] and folate biosynthesis [16], amino acid metabolism [17], sphingolipid [18] and phospholipid homeostasis [19], and mitochondrial respiration [20,21], all of which are essential for fungal viability and pathogenesis. Systematic characterization of such pathways in *C. auris* has lagged behind that of other pathogenic fungi, such as *C. albicans*, in part due to limited genetic tractability [22].

The polyamines putrescine, spermidine, and spermine are small polycationic metabolites that are essential for cellular growth, translation, chromatin organization, and stress adaptation [23,24,25,26,27]. Their intracellular levels are controlled by a conserved biosynthetic pathway, disruption of which leads to severe growth defects in many eukaryotes. In addition, studies in yeast, flies, nematodes, mice and humans have shown that spermidine induces autophagy and extends lifespan [28].

**Figure 1:**
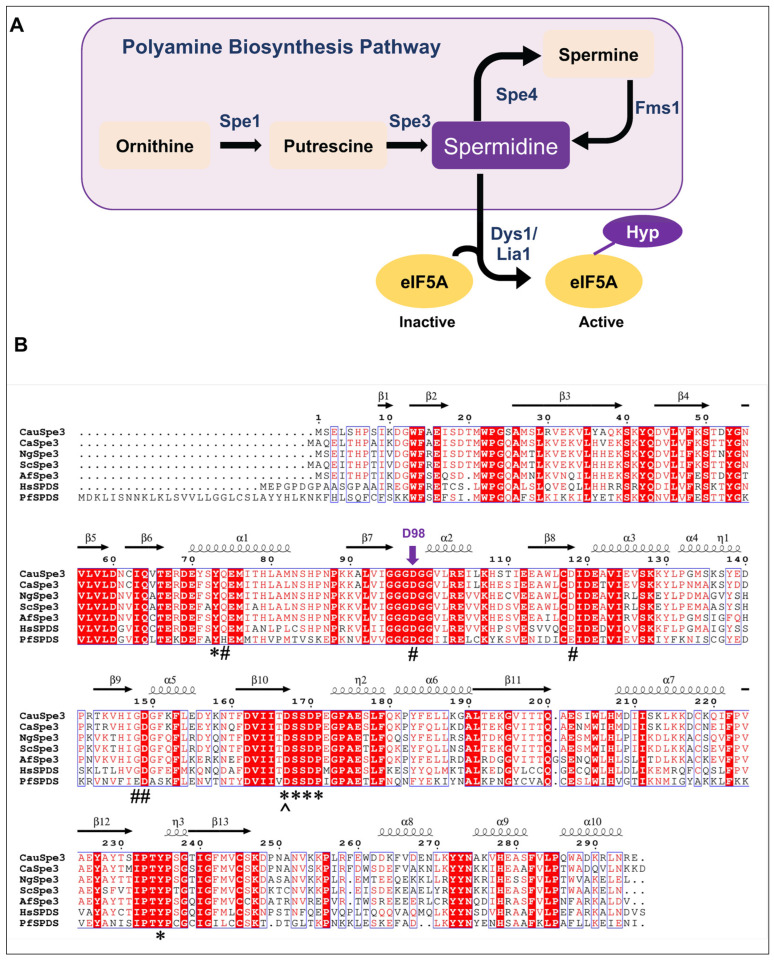
Polyamine-dependent metabolic network in fungi. (**A**) Polyamine biosynthesis. Ornithine is converted to putrescine by Spe1 (ornithine decarboxylase). Putrescine is aminopropylated by spermidine synthase, Spe3, using decarboxylated S-adenosylmethionine (dc-SAM) to generate spermidine, with 5′-methylthioadenosine (MTA) produced as a byproduct. Spermidine is further converted to spermine by Spe4 using dc-SAM as the aminopropyl donor. Spermidine is required for hypusination of eIF5A. Spermine is oxidatively converted by polyamine oxidase, Fms1p, to spermidine and 3-aminopropanal. (**B**) Sequence alignment of spermidine synthases. Multiple sequence alignment of C. auris spermidine synthase (CauSpe3) with representative orthologs from *Candida albicans* (XP_714840), *Nakaseomyces glabrata* (XP_445476) and *Aspergillus fumigatus* (XP_752719), *S. cerevisiae* (NP_015394), human (NP_003123), and *Plasmodium falciparum* (XP_001347972). Alignment was generated using MultAlin version 5.4.1 [40] and displayed with ESPrit [41]. Conserved catalytic residues are indicated, including Q43, Q74, D98, D119, D149, and G150 as dc-SAM binding residues (#); Y73, the DSSD motif (167–170), and Y235 as putrescine binding residues (*); and D167 as the proton acceptor (^). Consensus levels are indicated as follows: residues with ≥90% conservation are highlighted in red. Secondary structure elements of CauSpe3 are shown above the alignment.

A central step in polyamine biosynthesis is catalyzed by aminopropyltransferases (APTs), which transfer an aminopropyl group from decarboxylated S-adenosylmethionine (dc-SAM) to putrescine or spermidine, producing spermidine or spermine, respectively [29,30] (Figure 1A). In *S. cerevisiae*, spermidine synthase, Spe3, plays a central role in polyamine homeostasis and is essential for growth under conditions where exogenous polyamines or related metabolites, including spermidine, spermine, or pantothenic acid, are limiting [31].

Despite the therapeutic potential of targeting polyamine biosynthesis enzymes and downstream steps such as those controlling protein translation through eIF5A hypusination (Figure 1A), progress has been hindered by the lack of sensitive and scalable assays to directly measure enzyme activity to identify non-analog and novel inhibitors [31,32,33,34,35]. We recently addressed this limitation by developing DAB-APT, the first fluorescence-based assay for quantifying APT-catalyzed polyamine biosynthesis [36,37,38]. This assay exploits the reaction of polyamines with 1,2-diacetyl benzene (DAB) to generate fluorescent conjugates, with signal intensity correlating with polyamine chain length [36,37,38]. DAB-APT has been rigorously validated using bona fide, previously characterized, APT enzymes from *S. cerevisiae* and *Plasmodium falciparum*, confirmed by means of mass spectrometry and thin-layer chromatography, and optimized for kinetic analysis and high-throughput chemical screening [36,37,38].

Importantly, recent studies in *C. albicans* demonstrate that disruption of polyamine biosynthesis and uptake profoundly impairs virulence-associated traits, including morphogenesis and host adaptation [39]. Thus, defining the biochemical properties of CauSpe3 in *C. auris* provides not only direct validation of this pathway in this pathogen, but also establishes a foundation for target-based antifungal discovery in a species where metabolic vulnerabilities remain poorly defined.

## 2. Materials and Methods

### 2.1. Materials

*C. auris* spermidine synthase was codon-optimized for expression in both *S. cerevisiae* and *E. coli*, chemically synthesized and cloned into pMAL-c4x-1-H(RBS) plasmid by GenScript (Piscataway, NJ, USA). Putrescine (P5780-5G) and 1,2-diacetylbenzene (242039-100MG) were purchased from Millipore Sigma (St. Louis, MO, USA). Dc-SAM was purchased from BOC Sciences (Shirley, NY, USA). β-mercaptoethanol (1610710) was purchased from Bio-Rad (Hercules, CA, USA).

### 2.2. Expression and Purification of MBP-Fused CauSpe3

The plasmid encoding MBP-tagged CauSpe3 (CauSpe3–pMAL-c4x) was transformed into *E. coli Rosetta (DE3)* competent cells (Novagen) for expression according to the manufacturer’s protocol. Transformants were cultured in Luria–Bertani (LB) medium [42] supplemented with ampicillin (50 µg/mL) at 37 °C until reaching the mid-log phase (OD_600_ ~0.6), induced with 0.5 mM isopropyl β-D-thiogalactopyranoside (IPTG), and incubated at 16 °C for 12–16 h. Cells were transferred to 50 mL polypropylene conical tubes and harvested via centrifugation at 5000× *g* for 10 min at 4 °C. The cell pellet was resuspended in lysis buffer containing 25 mM Tris-HCl (pH 8.0), 500 mM NaCl, 0.5% glycerol, protease inhibitor cocktail, and 0.002% CHAPS. Cell disruption was performed via sonication using a Sonifier system (Branson Ultrasonics, Danbury, CT, USA) with 2 s pulses at 25% amplitude interspersed with 2 s cooling intervals on ice (total sonication time, 2.5 min), and cellular debris was removed by centrifugation at 16,000× *g* for 20 min. The supernatant was applied to amylose resin equilibrated in binding buffer (20 mM Tris-HCl, 200 mM NaCl, 1 mM EDTA, 1 mM DTT) at 4 °C with gentle agitation. After thorough washing, the fusion protein was eluted with a binding buffer containing 10 mM maltose. Purity was evaluated via SDS–PAGE with Coomassie staining, and protein concentrations were determined spectrophotometrically.

### 2.3. Fluorescence-Based DAB-Spe3 Assay

The Spe3 enzyme reaction and subsequent quantification of spermidine using the 1,2 diacetylbenzene/β-mercaptoethanol detection system were carried out in two sequential steps [36,38,43]. In the initial step, recombinant CauSpe3 and the mutant variant CauSpe3^mut^ were assayed under the standard reaction conditions in a total volume of 100 µL and incubated at 37 °C. Reactions were incubated at 37 °C. At specified intervals (0, 20, 40, and 60 min), 17.5 µL aliquots were withdrawn and the reactions were terminated by heating at 95 °C for 5 min. Samples were then cooled on ice prior to fluorescence-based analysis. For detection, 17.5 µL of each heat-inactivated reaction was mixed with 17.5 µL distilled water and 85 µL of detection solution containing 1.75 mM βME, 20 mM sodium buffer (pH 9.6), 0.22 mM potassium phosphate, and 1.48 mM 1,2-diacetylbenzene. The mixtures were transferred to a black 96-well clear-bottom plate (265301, ThermoFisher Scientific, Waltham, MA, USA) and incubated at room temperature for 60 min to allow fluorophore development. Fluorescence was recorded using a plate reader (Synergy H1, Agilent, Santa Clara, CA, USA) with excitation at 364 nm and emission at 425 nm. Data were processed and analyzed using Prism 10 software (GraphPad, Boston, MA, USA).

### 2.4. Determination of Spe3 Activity and Reaction Velocity

Fluorescence signals generated in the DAB-based Spe3 assay were processed as previously described for polyamine detection assays [36,37,38]. Signals were first background-corrected by subtracting values obtained from mock reactions containing heat-denatured enzyme without putrescine. To eliminate contributions from nonenzymatic fluorescence, particularly that arising from direct interactions between assay components and detection reagents, an additional correction was applied by subtracting fluorescence measured from heat-inactivated enzyme samples incubated with putrescine and dc-SAM. In kinetic analyses, normalized fluorescence readings were translated into micromolar spermidine concentrations using the corresponding 100% spermidine reference matched to each putrescine concentration. Velocities were calculated and reported as nmol of spermidine produced per µg protein per minute [38]. Reaction rates were plotted against ornithine concentration, and kinetic parameters were obtained by fitting the data to the sigmoidal (Hill equation) model using nonlinear regression in GraphPad Prism 10 [36].

### 2.5. Analysis of Spermidine and Putrescine by Thin-Layer Chromatography

Formation of spermidine from putrescine following the Spe3 reaction was verified via thin-layer chromatography (TLC) on Silica 60 plates (Merck; 500 mm) using a solvent system consisting of n-butanol–acetic acid–pyridine–water (3:3:2:1, *v*/*v*/*v*/*v*) [36]. Spermidine and putrescine were detected by spraying the plates with ninhydrin solution (0.2% in ethanol/acetic acid, 99.5/0.5) and heating at 110 °C for 5 min.

### 2.6. Determination of Spe3 Activity Using LC-MS

The polyamine standards were prepared at a concentration range of 25–2500 ng/mL, and quality controls (QCs) were prepared at a concentration of 100 and 500 ng/mL. CauSpe3 catalyzed enzyme reactions designated for LC-MS analysis of enzyme product (spermidine) were resuspended in an equal volume of sodium phosphate buffer (pH 7.2) and incubated at 4 °C for 3 h with intermittent mixing. Following incubation, three volumes of ice-cold acetonitrile were added to each reaction, and the mixtures were centrifuged at 2100× *g* for 15 min at 4 °C. The resulting supernatants were collected and analyzed by means of mass spectrometry to detect polyamine peaks. A 5 µL aliquot of each sample was injected into the LC-MS system for analysis. LC-MS analysis was performed using an API 4000 QTrap^®^ mass spectrometer (Applied Biosystems Sciex, Toronto, ON, Canada) coupled to an Agilent HP1200 HPLC system (Agilent Technologies, Santa Clara, CA, USA). Data acquisition and processing were carried out using Analyst 1.7 software. Quantification of putrescine and spermidine was achieved using a targeted Multiple Reaction Monitoring (MRM) approach. Chromatographic separation was achieved on an Agilent Eclipse XDB-C18 column (3.5 µm, 2.1 × 100 mm) equipped with a C18 guard column, maintained at 50 °C. The mobile phase consisted of 0.1% formic acid in water (solvent A) and 0.1% formic acid in acetonitrile (solvent B). The gradient started at 98% A for 0.2 min, increased to 30% B over 4.3 min, to 50% B in 1.5 min, and then to 85% B within 0.5 min. The composition was held at 85% B for 1.5 min, returned to 2% B in 0.5 min, and equilibrated for 3.5 min before the next injection. The mass spectrometer was operated in positive electrospray ionization (ESI) mode. MRM transitions monitored for putrescine and spermidine were 89.1/72.0, 133.1/116.2, 146.2/72.0, and 203.2/129.1, respectively. Declustering potentials were 48, 45, 35, and 60 eV; entrance potentials were 5, 5, 5, and 9 eV; collision cell exit potentials were 10, 7, 10, and 10 eV; and collision energies were 14, 13, 20, and 17 eV, respectively. The ion spray voltage was 5500 V, with a source temperature of 400 °C. Ion source gases 1 and 2 were maintained at 50 psi, while the curtain and collision-activated dissociation (CAD) gases were set to 15 psi and high mode, respectively.

### 2.7. Transformation of the Plasmids Harboring SPE3 Genes into S. cerevisiae

The *S. cerevisiae spe3Δ::KanMX* (BY4741) strain was transformed with plasmid constructs (pBEVY-U empty vector, pBEVY-U–CauSPE3, pBEVY-U–CauSPE3^D98A^, and p5392–ScSPE3) using the Yeastmaker™ Yeast Transformation System 2 (Clontech Laboratories, San Jose, CA, USA) according to the manufacturer’s instructions. Briefly, cells were grown to mid-log phase and rendered competent using a lithium acetate/polyethylene glycol (LiAc/PEG)-based method. Plasmid DNA was mixed with denatured carrier DNA and incubated with competent cells in PEG/LiAc solution, followed by heat shock at 42 °C in the presence of DMSO. Following transformation, cells were plated onto vitamin-defined pantothenate-free medium (VFM) lacking uracil [10] and supplemented with spermidine and pantothenic acid to support the growth of the *spe3Δ* strain harboring pBEBY-U empty vector or pBEVY-U–CauSPE3^D98A^. Transformants were selected after incubation at 30 °C and used for subsequent growth assays as described below.

### 2.8. Yeast Growth Assays

Yeast transformants (*spe3Δ::KanMX*/BY4741 background) generated as described above were grown overnight at 30 °C in vitamin-defined pantothenate-free synthetic medium (VFM) supplemented with spermidine and pantothenic acid [10]. Cells were harvested (700× *g*, 5 min, 4 °C), washed with sterile water, and resuspended in fresh VFM at OD_600_ = 0.5. For spotting assays, 10-fold serial dilutions were prepared and 5 µL aliquots were spotted onto VFM agar plates containing either no supplementation or supplemented with putrescine (250 µM), spermidine (100 µM), or spermine (250 µM). Plates were incubated at 30 °C, and growth was documented every 24 h using a ChemiDoc MP imaging system (Bio-Rad, Hercules, CA, USA). VFM consisted of 2% glucose and 6.7 g/L yeast nitrogen base with ammonium sulfate and without vitamins (MP Biomedicals, Irvine, CA, USA), supplemented with a URA dropout amino acid mixture (MP Biomedicals) and vitamins (excluding pantothenic acid) as follows: niacin (nicotinic acid), 0.4 mg/L; p-aminobenzoic acid, 0.2 mg/L; pyridoxine hydrochloride, 0.4 mg/L; riboflavin, 0.2 mg/L; thiamine hydrochloride, 0.4 mg/L; folic acid, 2 µg/L; biotin, 2 µg/L; and inositol, 2 mg/L. For liquid growth assays, strains were pre-grown as described above, washed, and diluted into VFM supplemented with either no supplementation or 0.5 mM putrescine, spermidine, or spermine. Growth was monitored spectrophotometrically by measuring OD_600_ using a BioTek microplate reader (Agilent, Santa Clara, CA, USA) at multiple time points.

## 3. Results

### 3.1. Identification of C. auris Spe3

To identify spermidine synthases from fungal pathogens, the *Saccharomyces cerevisiae* Spe3 protein was used as a query to mine publicly available fungal genomic databases. Candidate homologs were retrieved based on sequence similarity and domain architecture. Multiple sequence alignment demonstrated strong conservation across species (Figure 1B). This search identified a single protein, ID: KAK8443457.1, here referred to as CauSpe3, as the predicted spermidine synthase in the *C. auris* proteome. CauSpe3 shares 74% identity and 85% similarity with spermidine synthases from *S. cerevisiae*. Structural and biochemical studies of ScSpe3 enzyme have defined key catalytic residues required for dc-SAM binding, putrescine binding, and proton transfer. Consistent with these studies, conserved catalytic residues in CauSpe3 include Q43, Q74, D98, D119, D149, and G150 involved in dc-SAM binding; Y73, the DSSD motif (residues 167–170), and Y235 involved in putrescine binding; and D167, which functions as the catalytic proton acceptor. Notably, the aspartate at position 98 (D98), previously demonstrated to be essential for catalytic activity in other Spe3 enzymes [44], is fully conserved in CauSpe3 (Figure 1B).

**Figure 2:**
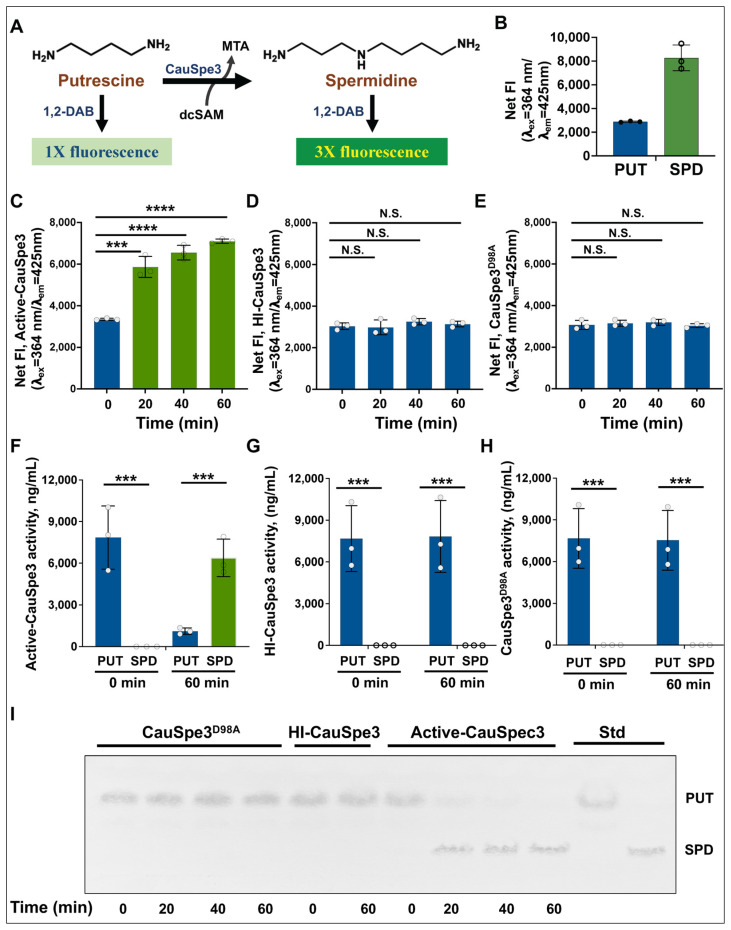
Biochemical characterization of CauSpe3 using a DAB-based fluorescence assay (**A**) Schematic representation of the DAB-based fluorescence assay. Spermidine formation from putrescine in the presence of dc-SAM is detected through the formation of fluorescent DAB adducts (λ_ex_ = 364 nm; λ_em_ = 425 nm). (**B**) Fluorescence intensity of putrescine and spermidine after reaction with DAB and β-mercaptoethanol. Spermidine produced ~3-fold higher fluorescence than putrescine. (**C**) CauSpe3-catalyzed reactions were conducted with putrescine and dc-SAM for the indicated time periods. At each time point, reactions were terminated prior to addition of DAB detection buffer, and reaction products were subsequently derivatized to generate fluorescent adducts (λ_ex_ = 364 nm; λ_em_ = 425 nm) as described in the Methods. A time-dependent increase in fluorescence was observed in reactions containing purified active CauSpe3, putrescine, and dc-SAM, consistent with spermidine formation. (**D**,**E**) Control reactions containing heat-inactivated (HI) CauSpe3 (**D**) or the catalytic mutant CauSpe3^D98A^ (**E**) show no significant increase in fluorescence, demonstrating dependence on enzymatic activity. (**F**–**H**) SPDS reaction products were independently analyzed by means of liquid chromatography–mass spectrometry (LC–MS). Reaction samples collected at 0 and 60 min were subjected to LC–MS analysis to monitor substrate consumption and product formation. In reactions containing active-CauSpe3 (**F**), a decrease in putrescine signal accompanied by the appearance of a spermidine peak was observed at 60 min, confirming enzymatic conversion. In contrast, reactions containing HI-CauSpe3 (**G**) or the catalytically inactive mutant CauSpe3^D98A^ (**H**) showed no detectable spermidine formation and no significant reduction in putrescine levels. Statistical significance was determined using Student’s t-test. *** *p* < 0.001, **** *p* < 0.0001; N.S., not significant. (**I**) Thin-layer chromatography (TLC) analysis of SPDS reaction products. Samples collected at the indicated time points were resolved on silica plates and visualized by means of ninhydrin staining. An increase in the spermidine (SPD) spot intensity was observed in a time-dependent manner in reactions containing active-CauSpe3, consistent with the fluorescence increase detected in panel (**B**). No spermidine formation was observed in reactions containing HI-CauSpe3 or CauSpe3^D98A^ mutant enzyme.

### 3.2. Characterization of CauSpe3 Activity Using a DAB-Based Fluorescence Assay

**Figure 3:**
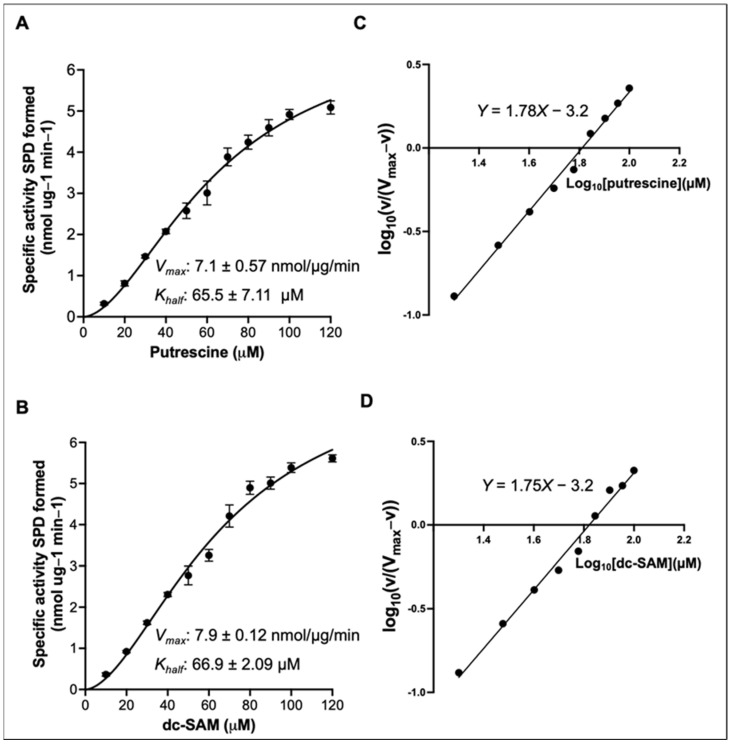
Determination of kinetic parameters of CauSpe3 using the DAB-based fluorescence assay. (**A**) Sigmoidal (Hill equation) analysis of CauSpe3 activity as a function of putrescine concentration. Enzymatic reactions were performed with varying concentrations (0–120 µM) of putrescine while maintaining dc-SAM at saturating levels (200 µM). Reaction velocities were calculated from spermidine formation measured using the DAB-based fluorescence assay (λ_ex_ = 364 nm; λ_em_ = 425 nm). Nonlinear regression fitting using GraphPad Prism 10 yielded a K_half_ of 65.5 ± 7.11 µM and a *V_max_* of 7.1 ± 0.57 nmol·µg^−1^·min^−1^ with a Hill coefficient (h) of 1.78. (**B**) Sigmoidal (Hill equation) analysis of CauSpe3 activity as a function of dc-SAM concentration. Reactions were performed with varying concentrations of dc-SAM (0–120 µM) while maintaining putrescine at saturating levels (200 µM). Nonlinear regression analysis yielded a K_half_ of 66.9± 2.09 µM and a *V_max_* of 7.9 ± 0.12 nmol·µg^−1^·min^−1^, with a Hill coefficient (h) of 1.75. (**C**) Hill plot derived from the putrescine-dependent kinetics shown in panel A. Data were transformed as log_10_(v/(Vmax − v)) versus log_10_[putrescine] (µM) using Vmax from nonlinear regression. Linear fitting over the range 20–100 µM confirmed the Hill coefficient obtained in panel A (R^2^ = 0.994). (**D**) Hill plot derived from the dc-SAM-dependent kinetics shown in panel B. Data were transformed as log_10_(v/(Vmax − v)) versus log_10_[dc-SAM] (µM). Linear fitting over the range 20–100 µM confirmed the Hill coefficient obtained in panel B (R^2^ = 0.989). Data represent the mean of three independent experiments, each performed in triplicate.

To evaluate the catalytic activity of CauSpe3, a codon-optimized gene was synthesized and cloned into pMAL-c4X for bacterial expression as an MBP fusion protein and subsequent purification on an amylose column (Figure S1). The enzyme activity of CauSpe3 was measured using the DAB-APT fluorescence assay (Figure 2A). This assay detects spermidine formation through differential fluorescence generated by DAB adduct formation with polyamines [36,37,38]. Incubation of purified CauSpe3 with putrescine and dc-SAM resulted in a time-dependent increase in net fluorescence (λ_ex_ = 364 nm; λ_em_ = 425 nm) over 60 min (Figure 2B). In contrast, no significant fluorescence increase was observed in reactions containing heat-inactivated enzyme or the catalytic mutant CauSpe3^D98A^ (Figure 3C,D), indicating that signal generation depends on active enzyme. LC–MS analysis of reaction products collected at 0 and 60 min confirmed conversion of putrescine to spermidine by active CauSpe3, whereas no product formation was detected in reactions containing heat-inactivated or CauSpe3^D98A^ mutant enzyme (Figure 2E–G). Thin-layer chromatography further validated spermidine production in reactions containing active CauSpe3 (Figure 2H). These results validate that the DAB-based fluorescence assay accurately reflects CauSpe3 enzymatic activity and provides a reliable method for measuring spermidine synthase activity *in vitro*.

### 3.3. Kinetic Characterization of CauSpe3

Steady-state kinetic parameters of CauSpe3 were determined by measuring enzymatic activity using the DAB-based fluorescence assay. Reaction velocities were calculated from spermidine formation detected at 364/425 nm. Enzyme activity was measured at varying concentrations of putrescine or dc-SAM while maintaining the co-substrate at saturating levels (Figure 3A,B). CauSpe3 exhibited sigmoidal kinetics with respect to both substrates. When putrescine concentration was varied, the enzyme exhibited a *V_max_* of 7.1 ± 0.57 nmol·µg^−1^·min^−1^ and a *K_half_* of 65.5 ± 7.11 µM, and a Hill coefficient of 1.78 (95% CI: 1.61–1.96) (Figure 3A). When dc-SAM concentration was varied, a *V_max_* of 7.9 ± 0.12 nmol·µg^−1^·min^−1^ and a *K_half_* of 66.9 ± 2.09 µM, and a Hill coefficient of 1.75 (95% CI: 1.58–1.93) were obtained (Figure 3B). These values indicate moderate positive cooperativity for both substrates. Hill plot analysis of the transformed data (Figure 3C,D) yielded slopes consistent with the Hill coefficients obtained from nonlinear regression, further supporting the sigmoidal nature of the observed kinetics. Such behavior is consistent with the two-substrate nature of spermidine synthase and has been reported for orthologous enzymes from *S. cerevisiae* and *P. falciparum*. The kinetic parameters of CauSpe3 are comparable to those reported for *S. cerevisiae* Spe3 [36], supporting conservation of a catalytically constrained and potentially druggable enzymatic step in *C. auris* polyamine metabolism.

### 3.4. Biological Activity of CauSpe3 in Yeast Complementation Assays

**Figure 4:**
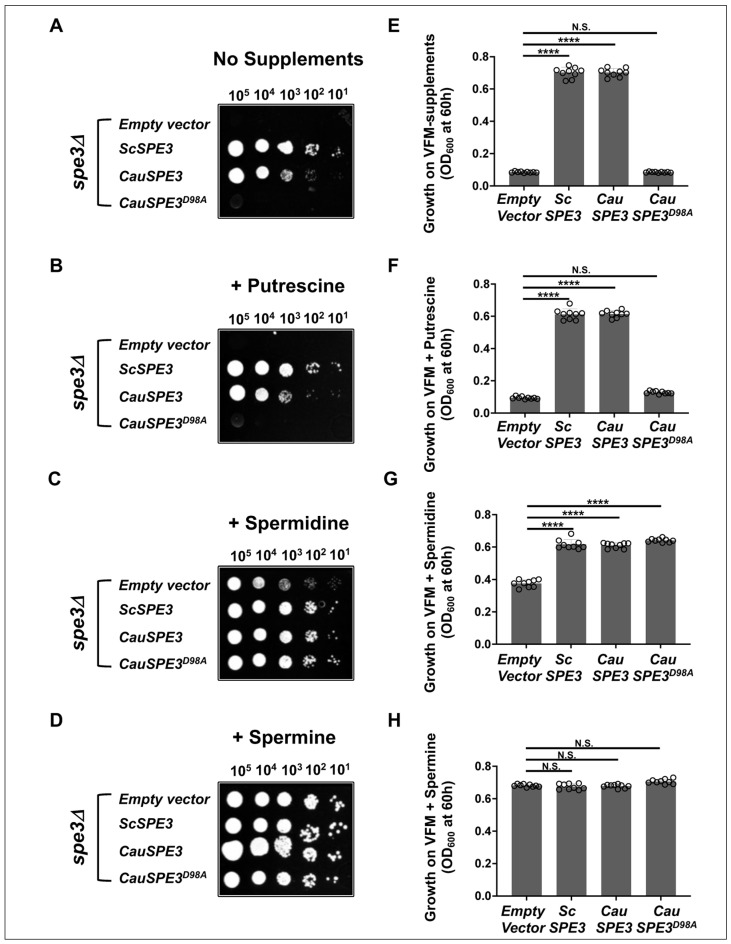
Biological activity of CauSpe3 in yeast complementation assays. (**A**–**D**) Serial dilution growth assay of S. cerevisiae *spe3Δ* strains transformed with empty pBEVY-U vector, ScSpe3 (positive control), CauSpe3 (wild-type), or CauSpe3^D98A^ (catalytic mutant). Cells were serially diluted (10-fold) and spotted onto selective vitamin-defined synthetic media under the indicated supplementation conditions of no addition (**A**), putrescine (PUT, 0.25 mM) (**B**), spermidine (SPD, 0.1 mM) (**C**), or spermine (SPM, 0.25mM) (**D**) and incubated at 30 °C. (**E**–**H**) Liquid growth assay under supplementation with no addition (**E**), 0.5 mM putrescine (PUT) (F), spermidine (SPD) (**G**), or spermine (SPM) (**H**). Cells were inoculated into 100 µL of vitamin-defined synthetic liquid media supplemented as indicated at an initial density of 10 cells per µL and incubated at 30 °C or 60 h. Cell growth was monitored by measuring OD_600_. Statistical significance was determined using Student’s *t*-test. **** *p* < 0.0001; N.S., not significant.

To assess biological activity, codon-optimized CauSpe3 and the catalytically inactive mutant CauSpe3^D98A^ were cloned into the yeast expression vector pBEVY-U (*URA3* marker) [45] and introduced into an *S. cerevisiae spe3Δ* strain, lacking the *SPE3* gene, following selection on vitamin-free media (VFM) lacking uracil but supplemented with spermidine and pantothenic acid as described in the methods. As a control, the *spe3Δ* mutant was also transformed with pBEVY-U empty vector alone or expressing the *S. cerevisiae* wild-type *ScSPE3* gene. Due to the loss of the *SPE3* gene, the *spe3Δ* strain is auxotrophic for spermidine and requires either spermidine or spermine for survival. In serial 10-fold dilution assays on solid VFM plates, *spe3Δ* strains harboring either the empty vector or the CauSpe3^D98A^ mutant showed no detectable growth in VFM medium lacking supplements or supplemented with putrescine (Figure 4A). In contrast, expression of wild-type CauSpe3 or ScSpe3 restored growth in these media. Supplementation with spermidine or spermine rescued the growth of all strains. These findings were further corroborated in liquid growth assays (Figure 4B). These data indicate that CauSpe3 functions as a spermidine synthase *in vivo* and that the conserved D98 residue is required for its activity. Importantly, this complementation platform establishes a convenient genetic system for evaluating inhibitors targeting CauSpe3, as compounds that inhibit the enzyme would be expected to phenocopy the spermidine auxotrophy observed in the spe3Δ strain. Beyond confirming *in vivo* enzymatic activity, this complementation platform establishes a genetically tractable surrogate system for evaluating CauSpe3 inhibitors and defining pathway vulnerability under controlled polyamine conditions. This is particularly valuable for *C. auris*, where direct genetic interrogation of metabolic essentiality remains comparatively limited.

## 4. Discussion

In this study, we report the first biochemical and functional characterization of spermidine synthase from the emerging fungal pathogen *C. auris*. Biochemical analyses of recombinant CauSpe3 demonstrate that it efficiently catalyzes the conversion of putrescine to spermidine in the presence of decarboxylated S-adenosylmethionine. This enzymatic activity is further supported by heterologous complementation in *S. cerevisiae*, showing that the *C. auris SPE3* gene encodes a functional aminopropyltransferase capable of restoring spermidine biosynthesis in a *spe3∆* strain. Together, these findings provide direct experimental validation of spermidine biosynthesis in *C. auris*, a pathway that until now had been inferred solely from genomic annotation. Given the limited knowledge about polyamine metabolism and related enzymes in *C. auris*, this work defines one of the first experimentally anchored nodes in the pathogen’s core polyamine network and provides a framework for linking this pathway to persistence, stress adaptation, and antifungal resistance.

This work was made possible through the application of the recently developed fluorescence-based DAB-APT assay to measure the activity of aminopropyltransferases [36,37,38]. Traditional methods for measuring polyamine biosynthesis have relied on radiolabeled substrates, low-throughput chromatographic techniques, or indirect readouts that limit scalability and mechanistic insight [31,32,33,34,35]. In contrast, the DAB-APT assay enables sensitive, quantitative, and direct measurement of aminopropyltransferase activity, facilitating kinetic analysis and rigorous validation using appropriate enzymatic controls [36,37,38].

The complete loss of activity observed with a catalytic-site mutant (CauSpe3^D98A^) and with heat-inactivated CauSpe3, together with independent confirmation by thin-layer chromatography, underscores both the specificity of the assay and the catalytic competence of the recombinant enzyme. Importantly, this study represents the first application of DAB-APT to a fungal pathogen of high clinical relevance, highlighting its utility beyond model organisms and parasites.

Polyamine metabolism is highly conserved across eukaryotes and is essential for fundamental cellular processes including translation, chromatin organization, and stress adaptation [23,24,25,26,27]. In fungi, perturbation of polyamine homeostasis impairs growth, biofilm formation, stress tolerance, and virulence-related traits, underscoring the biological importance of this pathway [46,47,48,49]. Spermidine occupies a central position in cell physiology, serving not only as a precursor for spermine synthesis but also as a required substrate for hypusination of eukaryotic initiation factor 5A [32,37,50,51]. The apparent positive cooperativity observed for both putrescine and dc-SAM in CauSpe3 may provide a mechanism for buffering spermidine synthesis against fluctuations in intracellular precursor availability, enabling the pathway to respond sharply once substrate pools exceed a threshold concentration. The biochemical validation of CauSpe3 therefore establishes a key enzymatic step in *C. auris* polyamine metabolism and provides a foundation for dissecting how this pathway contributes to fungal physiology under diverse environmental conditions. Consistent with this broader role, CauSpe3 is upregulated in *C. auris* biofilms [52], further supporting the idea that polyamine biosynthesis contributes to biofilm-associated growth, stress adaptation, and antifungal tolerance.

Notwithstanding its conservation across fungi, the extent to which spermidine synthase represents a viable target in *C. auris* remains to be determined. In *S. cerevisiae*, loss of Spe3 is conditionally lethal, with viability dependent on the availability of exogenous polyamines or pantothenic acid, underscoring the metabolic plasticity of the polyamine biosynthetic network and emphasizing the need for rigorous genetic and pharmacological target-validation studies to accurately define pathway vulnerability beyond sequence conservation alone. Genetic studies in *C. albicans*, showed that disruption of polyamine synthesis in combination with transport markedly impairs serum-induced hyphal differentiation and abolishes virulence in disseminated candidiasis models [39]. Polyamine levels have also been linked to yeast-to-filamentous transitions that are central to *C. albicans* virulence [53]. In *Aspergillus flavus*, deletion of spermidine synthase gene attenuates growth, secondary metabolite production, and virulence on host plants [54]. Whether *C. auris* can compensate for reduced polyamine biosynthesis through uptake of extracellular metabolites or whether *de novo* polyamine production is required for growth and virulence in this pathogen remains to be determined. While genetic and pharmacological validation studies remain to be performed, the biochemical data established here provides an essential starting point for further structural and chemical investigations. The availability of a high-throughput assay for CauSpe3 activity enables systematic evaluation of small-molecule inhibitors and structure–activity relationships *in vitro*. When combined with emerging genetic tools and well-defined *in vitro* growth models for *C. auris*, such studies will be critical for determining whether interference with polyamine biosynthesis is a viable strategy to identify novel antifungal drugs to counter the multidrug resistance profile of *C. auris* and the emergence of multidrug-resistant isolates of other species of fungal pathogens such as *C. albicans*, *Nakaseomyces glabrata* and *Aspergillus fumigatus*. More broadly, this work illustrates how enzyme-focused biochemical validation, coupled with enabling assay technologies, can accelerate metabolic characterization of high-priority fungal pathogens and inform future antifungal discovery efforts. The strong functional conservation with ScSpe3 provides evidence that *C. auris* retains a conserved and likely constrained dependence on spermidine biosynthesis that may be exploitable pharmacologically.

## 5. Conclusions

In conclusion, this study defines the functional and biochemical properties of CauSpe3 in *C. auris*. The quantitative DAB-based assay described herein enables detailed mechanistic analysis and provides a foundation for future efforts to identify inhibitors of polyamine biosynthesis.

## Data Availability

All data are contained in the article and the Supplemental Data Files.

## Supplementary Materials

The following supporting information can be downloaded at https://www.mdpi.com/article/10.3390/pathogens15040432/s1. Figure S1: SDS-PAGE analysis of purified MBP-CauSpe1 and MBP-CauSpe1^D98A^.

## Abbreviations

The following abbreviations are used in this manuscript:

CauSpe3	*C. auris* spermidine synthase
βME	β-mercatoethanol
1,2-DAB	1,2 diacetylbenzene
VFM	vitamin-defined pantothenate-free synthetic medium
SPDS	Spermidine synthase
APT	aminopropyltransferase
dc-SAM	decarboxylated S-adenosylmethionine
