# Supplementary material for: Functional and Biochemical Characterization of Spermidine Synthase CauSpe3 from Candidozyma auris
Source: Pathogens. 2026 Apr 16;15(4):432. doi: 10.3390/pathogens15040432 (PMC13118584; doi:10.3390/pathogens15040432)
Supplement: Supplementary file 1 [file pathogens-15-00432-s001.zip › pathogens-4211608-supplementary.pdf]

Figure S1

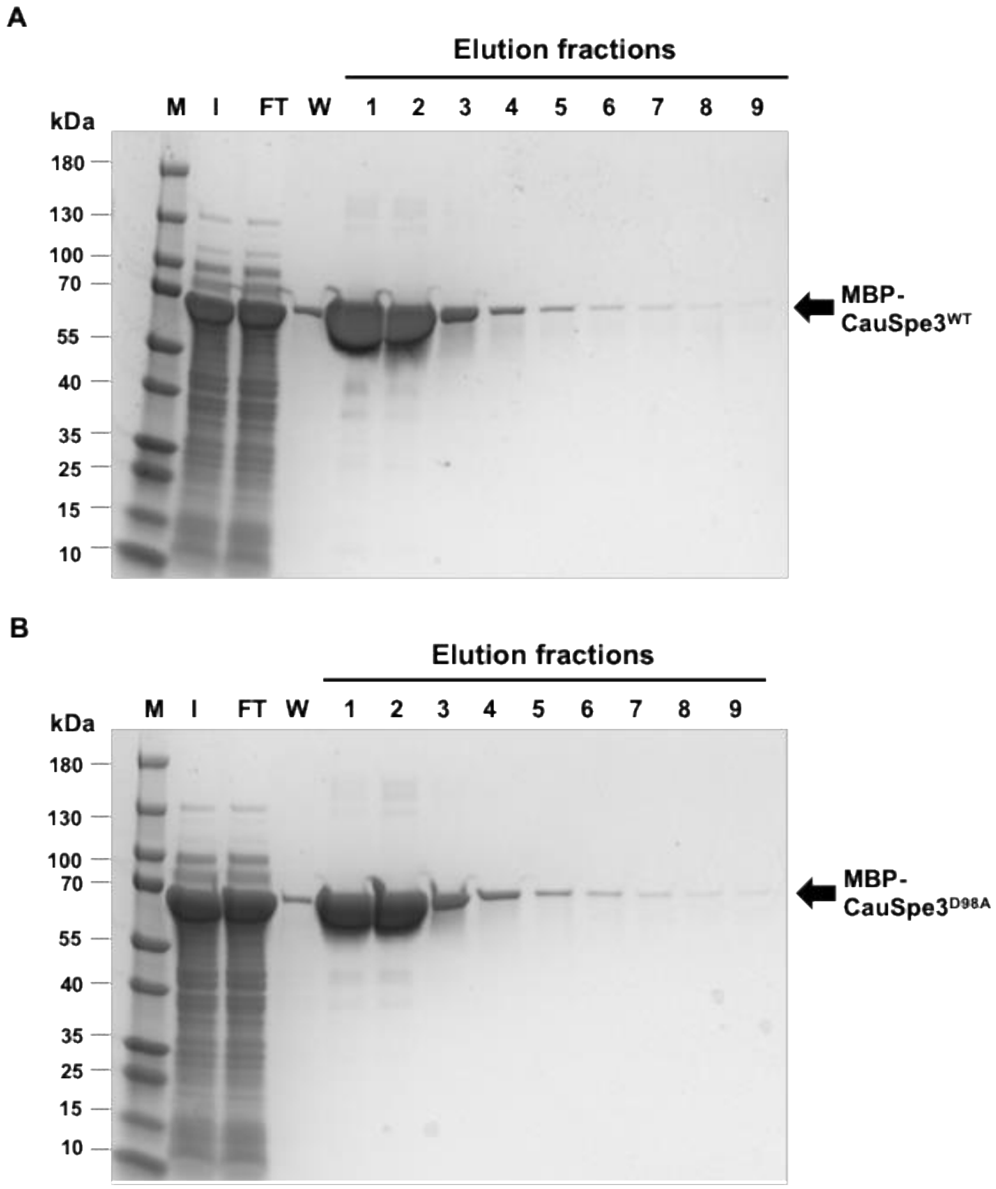

Figure S1. Expression and purification of MBP-tagged CauSpe3 wild type and active site mutant. A and B: SDS–PAGE analysis of affinity purification of recombinant MBP-CauSpe3^{WT} (A) and MBP-CauSpe3^{D98A} expressed in *E. coli*. Samples correspond to molecular weight marker (M), induced lysate (I), flow-through (FT), wash (W), and elution fractions (1–9). Elution fractions, 1-4, containing the target proteins were pooled and used for subsequent biochemical and enzymatic analyses.
